# Supplementary material for: A Workplace Health Promotion Program for a Predominantly Military Population: Associations with General Health, Mental Well-Being and Sustainable Employability
Source: Int J Environ Res Public Health. 2024 May 15;21(5):625. doi: 10.3390/ijerph21050625 (PMC11120656; doi:10.3390/ijerph21050625)
Supplement: Supplementary file 1 [file ijerph-21-00625-s001.zip › ijerph-2949932-supplementary.pdf]

International Journal of
Environmental Research and Public Health

MDPI

Supplementary Materials

Table S1. Multiple linear regression with difference scores in the health as dependent variable (M1–M0).

	Dif. General Health			Dif. BMI		
	β	t	*p*	β	t	*p*
Constant		2.576	0.010		−1.764	0.079
Sex (0 = male, 1 = female)	−0.084	−1.469	0.143	−0.029	−0.472	0.637
Age	−0.040	−0.694	0.488	0.069	1.107	0.269
Education level	−0.067	−1.192	0.234	0.042	0.691	0.490
Military department Central Staff (ref = Army Staff)	0.001	0.016	0.987	0.028	0.440	0.660
Military department Airforce Staff (ref = Army Staff)	0.081	1.405	0.161	−0.029	−0.462	0.645

Table S2. Multiple linear regression with difference scores in the mental well-being as dependent variable (M1–M0).

	Dif. Work Engagement			Dif. Burn-out		
	β	t	*p*	β	t	*p*
Constant		−1.660	0.098		−0.211	0.833
Sex (0 = male, 1 = female)	0.140	2.453	**0.015**	−0.028	−0.487	0.627
Age	0.069	1.225	0.221	0.011	0.190	0.849
Education level	0.079	1.403	0.162	−0.022	−0.388	0.698
Military department Central Staff (ref = Army Staff)	0.041	0.688	0.492	0.057	0.958	0.339
Military department Airforce Staff (ref = Army Staff)	0.070	1.217	0.224	−0.016	−0.277	0.782

Table S3. Multiple linear regression with difference scores in sustainable employability as dependent variable (M1–M0).

	Dif. Recovery after Work			Dif. Work-Home Balance			Dif. Work Ability		
	β	t	*p*	β	t	*p*	β	t	*p*
Constant		0.681	0.497		1.502	0.134		−0.864	0.388
Sex (0 = male, 1 = female)	0.073	1.266	0.206	0.045	0.779	0.436	0.066	1.149	0.251
Age	−0.017	−0.302	0.763	−0.068	−1.186	0.236	0.026	0.467	0.641
Education level	0.031	0.554	0.580	−0.043	−0.768	0.443	0.100	1.774	0.077
Military department Central Staff (ref = Army Staff)	−0.038	−0.636	0.525	−0.040	−0.668	0.505	−0.117	−1.975	0.050
Military department Airforce Staff (ref = Army Staff)	0.027	0.466	0.642	0.016	0.272	0.786	0.040	0.693	0.489
